# Multilocular lipoma of the left thumb of the hand: a case report

**DOI:** 10.1080/23320885.2021.1968302

**Published:** 2021-08-24

**Authors:** Ahmed Wafiq Wafa, Shabir Wani, Tuqa A. Alsinan, Sarah Alkhonizy

**Affiliations:** a Department of Plastic and Reconstructive Surgery, King Fahad Medical City, Riyadh, Saudi Arabia; b College of Medicine, Alfaisal University, Riyadh, Saudi Arabia

**Keywords:** Benign lipoma, lipoma of the thumb, soft-tissue lipoma, plastic surgery, hand surgery

## Abstract

Lipoma is a benign soft tissue tumor that is mostly found in the trunk, however, lipomas of the thumb are rarely reported, around 1% of cases. We report a case of a Saudi male aged 32-year-old who presented with a progressive left thumb swelling for a long time. The aim is to add more cases to the literature and to consider it as a differential.

## Background

As its name suggests, a lipoma is a benign adipocytic neoplasm that accounts for approximately 16% of soft tissue mesenchymal tumors [1–3]. It is usually presented as a painless, soft mass felt under the skin and most often encapsulated by a thin layer of fibrous tissue which can present in any part of the body [4]. The most common site of the neoplasm is the trunk, however, around 1% of cases, it can appear in the fingers with lipomas of the thumb specifically rarely reported [2,4]. Having a lipoma in the fingers is very rare, and the first patient ever was reported and documented in the year 1959 [5]. For management and treatment, they are usually not treated unless they cause pain due to their location or sometimes for cosmetic reasons [3,4].

## Case report

**Figure 1.**
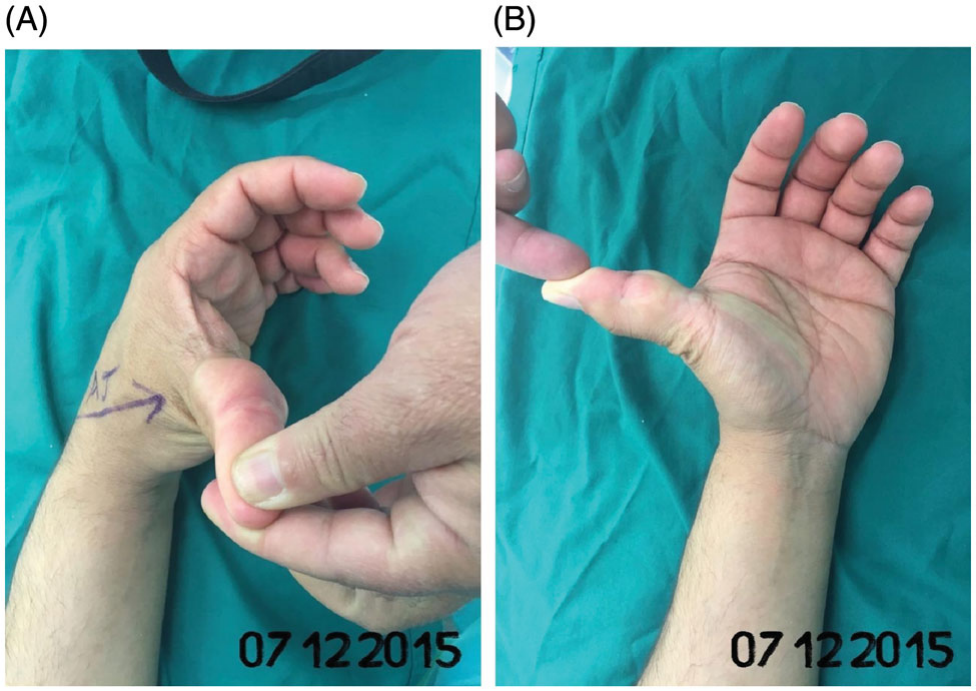
(A,B) A multilocular soft-tissue lipoma on the volar aspect of the left thumb.

A Saudi male patient aged 32-year-old right-handed government employee by profession presented with a progressive left thumb swelling that started 6 years ago. There were no other associated symptoms such as pain nor muscular atrophy. He was diagnosed with a multilocular soft-tissue lipoma on the volar aspect of the left thumb based on magnetic resonance imagining (MRI) findings, he is otherwise healthy and medically stable (Figure 1(A,B)). He doesn't smoke nor is he a second-hand smoker. There is no history of drug abuse, and travel history is insignificant. The rest of his history was unremarkable. Physical examinations were all within the normal limits.

**Figure 2.**
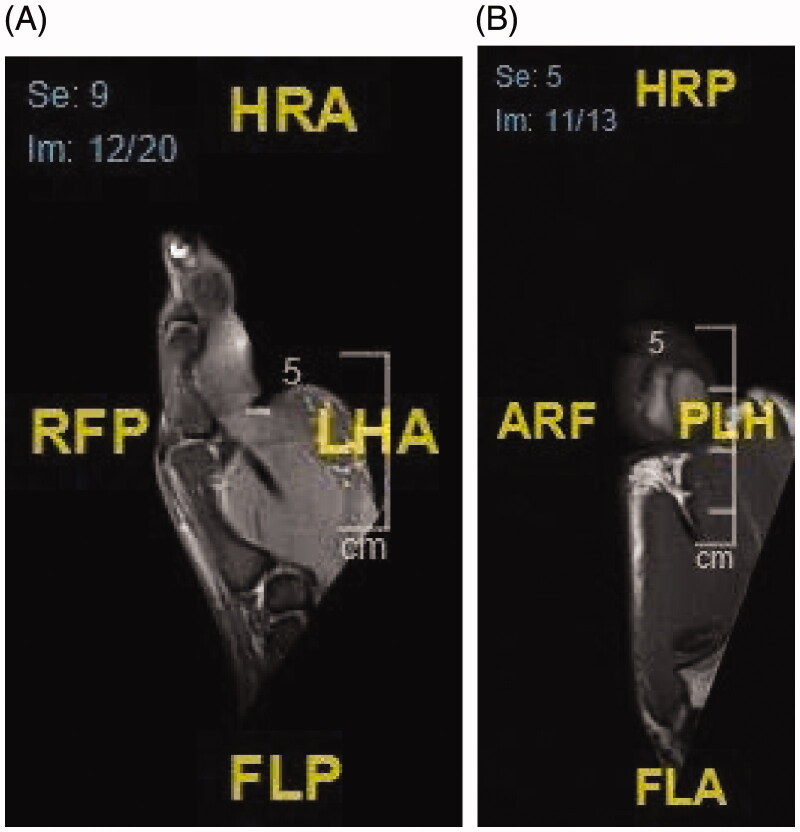
(A,B) The lipoma measures 2.2 × 2.8 × 4.3 cm in anteroposterior, transverse, and cranio-caudal diameters respectively.

**Figure 3.**
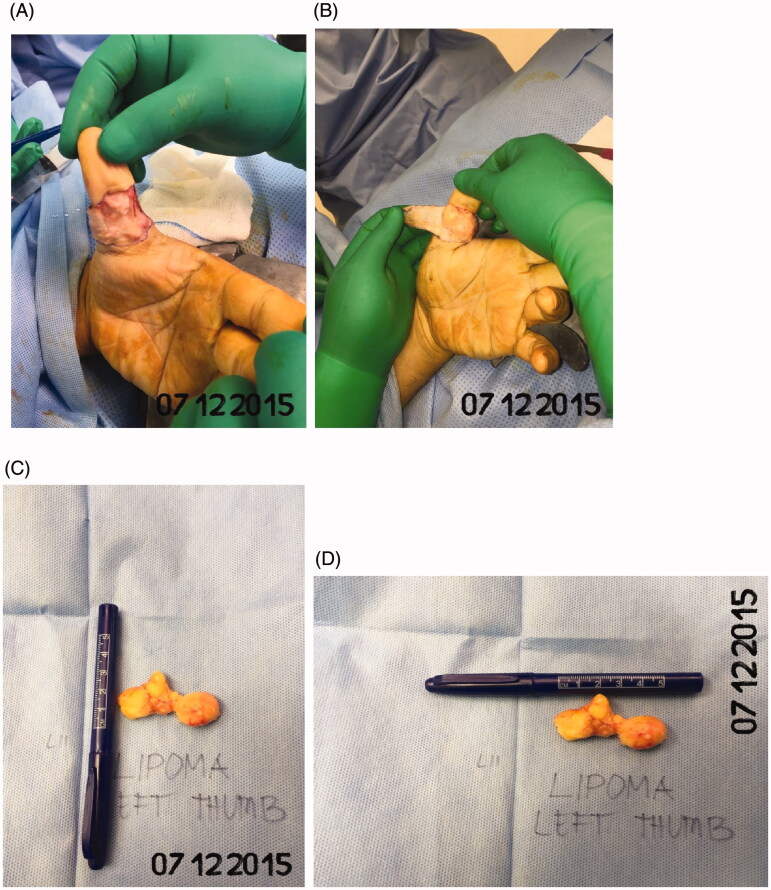
(A–D) A rectangular radially incision was made over the left thumb and the mass was excised completely.

For more investigations, the patient underwent several imaging workups. An MRI of the left thumb was ordered to confirm the findings, which showed a multilocular soft-tissue lesion along the volar aspect of the left thumb. The lesion extends from the level of the mid-distal phalanx to the first metacarpophalangeal joint. The lesion measures 2.2 × 2.8 × 4.3 cm in anteroposterior, transverse, and cranio-caudal diameters respectively. The lesion is surrounding the anterior, medial, and lateral borders of the flexor pollicis longus tendon with no deep extension to the tendon. After contrast administration, there is no definite enhancement. While the tendon of the flexor pollicis longus demonstrates a nonspecific abnormal signal intensity 1 cm above the base of the proximal phalanx. The remaining part of the tendon demonstrates normal signal intensity and thickness (Figure 2(A,B)). The patient was admitted to our institute for excision of the left thumb swelling. Under aseptic precautions and tourniquet control, a rectangular radially based incision was made over the left thumb over the thumb and the flap was raised in order to secure the neurovascular bundle. The mass was excised completely from the left thumb with no immediate or late complications (Figure 3(A–D)).

## Discussion

Lipomas are benign tumors that account for approximately 16% of soft tissue mesenchymal tumors [1,2]. They usually develop as a well-circumscribed mass felt under the skin. For diagnosis, a computed tomography (CT) scan and magnetic resonance imaging (MRI) is used in 71% of cases [2]. Lipoma can present in various locations and entities, rarely found in the fingers and specifically the thumb [2]. In most cases, lipomas are only observed clinically unless it's affecting the patients’ quality of life, in which surgical removal is commonly recommended [3,4]. Many cases were reported in the literature to document the rare entity of developing a benign lipoma in the hand, specifically of the thumb. Since the year 1979 in the United States, the first case in the literature was published by reporting a 9 year-old boy who presented with a mass in the dorsum of his right thumb [1]. Meanwhile, other two cases were reported to discuss the preferred surgical procedure in patients with infiltrating lipoma of the proper digital nerves, it was concluded that microdissection showed a good outcome by avoiding the interruption of the nerve in such cases [2]. On the other hand, several cases of soft-tissue tumors on the palmar aspect of the thumb either on the left or right thumb were reported in different countries and were added to the literature. It was concluded that surgical removal of the tumor showed an excellent outcome and considering a benign lipoma of the thumb as a differential diagnosis of similar swelling is necessary even if it is a rare entity [2,3,6,7].

Attached below is a table summarizing the previous similar cases.

**Table:** 

1979	Micheal Kalisman, A. Robert Beck	Lipoma of the dorsum of the thumb in a 9-year-old boy	The first reported case in the literature [6].
2006	P. Inaparthy Æ G. W. Southgate	Giant lipoma swelling of the right palm and the thenar eminence	This case reports the satisfactory and excellent outcome of surgical removal of the tumor [8].
2013	Hemalata T. Kamra, Santosh L. Munde	Lipoma swelling on the flexor aspect of proximal and distal phalanx of right thumb	This case reported the importance of considering uncommon lipomas of tendon sheath as a differential diagnosis in welling hands, especially of fingers [2].
2013	Luis Ramirez-Montaño , Ricardo Pacheco Lopez and Nicolas Sastre Ortiz	Giant tumor of the volar aspect of the third finger of the left hand	The case was reported due to its rare location and to rule out more common similar pathologies with varying prognoses [9].
2016	Johnny El Rayes, Roula Bou Sader, and Elie Saliba	Soft tissue swelling on the palmar aspect of the thumb	This case reported the first spindle cell lipoma in the thumb and to help considering such diagnosis of a similar swelling [1].
2018	Shogo Ebisudani, Ikuko Osugi, Kiichi Inagawa, Yoshinori Suzuki, Tomomi Kimura	Soft tissue swelling on the palmar aspect of the thumb	To their knowledge, this is the third case of spindle cell lipoma in the thumb in the literature [4].

## Conclusion

The aim of this case report is to add more to the literature by reporting more lipoma cases of the fingers, specifically the thumbs even if it is a rare entity and to consider it as a differential diagnosis, which eases ruling out malignant causes [2]. This article highlights the excellent outcome of cosmetic surgical removal in such patients [7].
